# Markers of hypercoagulability in CAD patients. Effects of single aspirin and clopidogrel treatment

**DOI:** 10.1186/1477-9560-10-12

**Published:** 2012-08-10

**Authors:** Vibeke Bratseth, Alf-Åge Pettersen, Trine B Opstad, Harald Arnesen, Ingebjørg Seljeflot

**Affiliations:** 1 Department of Cardiology, Center for Clinical Heart Research, Oslo University Hospital Ullevål, Pb 4956 Nydalen, Oslo, N-0424, Norway; 2 Center for Heart Failure Research, Oslo University Hospital, Oslo, Norway; 3 Faculty of Medicine, University of Oslo, Oslo, Norway

**Keywords:** Thrombin generation, Coronary artery disease, Hypercoagulability, Prothrombotic markers, The CAT assay, Endogenous thrombin potentiale, Antiplatelet treatment, Aspirin, Clopidogrel

## Abstract

**Background:**

Cardiovascular disease with disturbances in the haemostatic system, might lead to thrombotic complications with clinical manifestations like acute myocardial infarction (AMI) and stroke. Activation of the coagulation cascade with subsequent increased thrombin generation, characterizes a prothrombotic phenotype. In the present study we investigated whether prothrombotic markers were associated with risk factors and clinical subgroups in a cohort of patients with angiographically verified coronary artery disease (CAD). The patients were randomized to long-term treatment with the antiplatelet drugs aspirin or clopidogrel, and we further investigated the effect on hypercoagulability of such treatment for 1 year, of which limited data exists.

**Methods:**

Venous blood samples were collected in fasting condition between 08:00 and 10:30 am, at baseline when all patients were on aspirin therapy (n = 1001) and in 276 patients after 1 year follow-up on aspirin or clopidogrel. In vivo thrombin generation was assessed by prothrombin fragment 1 + 2 (F1+2) and D-dimer, and the endogenous thrombin potentiale (ETP) in the calibrated automated thrombogram (CAT) assay, representing ex vivo thrombin generation. In addition soluble tissue factor (sTF) and free- and total tissue factor pathway inhibitor (TFPI) were measured.

**Results:**

We found age to be significantly associated with F1+2 and D-dimer (β = 0.229 and β =0.417 respectively, p <0.001, both). Otherwise, only weak associations were found. F1+2 and D-dimer were higher in women compared to men (p <0.001 and p = 0.033, respectively). Smokers had elevated levels of ETP compared to non-smokers (p = 0.014). Additionally, patients on renin-angiotensin system (RAS) inhibition showed significantly higher levels of F1+2, compared to non-users (p = 0.013). Both aspirin and clopidogrel reduced levels of ETP after 12 months intervention (p = 0.003 and p <0.001, respectively) and the levels of F1+2 were significantly more reduced on aspirin compared to clopidogrel (p = 0.023).

**Conclusions:**

In the present population of stable CAD, we could demonstrate a more hypercoagulable profile among women, smokers and patients on RAS medication, assessed by the prothrombotic markers F1+2, D-dimer and ETP. Long-term antiplatelet treatment with aspirin alone seems to attenuate thrombin generation to a greater extent than with clopidogrel alone. The study is registered at http://www.clinicaltrials.gov: NCT00222261.

## Background

Cardiovascular disease (CVD) is a global burden in which thrombotic complications play a major role [1]. The underlying mechanism of CVD is mainly the atherosclerotic process, characterized by lipid accumulation in the subendothelium causing a low graded inflammatory lesion and changing the phenotype of the endothelium to a pro-inflammatory and pro-thrombotic state [2].

Disrupture of lesions expose atherogenic material into the arterial lumen. Platelets adhere, aggregate and become activated which may lead to thrombus formation. An occluded vessel may cause clinical manifestations like acute myocardial infarction (AMI) and stroke [3].

Expression of subendothelial tissue factor (TF) with subsequent activation of the coagulation cascade generates thrombin at the surface of activated platelets [2,3]. TF interacts with coagulation factor VII, forming an active complex which activates both FIX and FX. FXa is responsible for the proteolytic cleavage of prothrombin to thrombin, resulting in formation of prothrombin fragment 1+2 (F1+2) and further the formation of fibrin. TF is abundantly present in atherosclerotic plaques. In addition soluble TF (sTF) may be found on monocyte- or platelet- derived microparticles or sTF may be released from cells. The initiation of coagulation is regulated by tissue factor pathway inhibitor (TFPI) which inhibits both TF-FVIIa complex and activated FX [4,5]. The pro- and anticoagulant balance is essential in the prevention of thrombotic complications. Increased levels of the haemostatic markers sTF, F1+2 and D-dimer, the latter reflecting ongoing fibrin formation and degradation, have been shown in patients with CVD, whereas the role of TFPI in CVD is less clear [6].

Recently, the endogenous thrombin potentiale (ETP) measured by the calibrated automated thrombogram (CAT) assay, has been proposed to be an informative research tool to study the degree of hypercoagulability [7]. There are however, limited data on the association between CAT measures and CVD risk factors.

Patients with coronary artery disease (CAD) are routinely treated with antiplatelet medication, of which aspirin is most commonly used. Aspirin suppress the cyclooxygenase pathway in the arachidonic acid metabolism and has also been shown to possess anti-inflammatory properties probably by inactivating NF-kappa B [8,9]. Recently other anti-platelet drugs have become common in use, for instance clopidogrel, a thienopyridine, blocking the ADP-pathway. Since activated platelets act as a catalytic surface for thrombin generation, anti-platelet treatment in general, might have an anticoagulant effect, with a decline in generated thrombin [10]. However, limited knowledge of any effects on prothrombotic markers of clopidogrel exists.

The aim of the present study was to investigate whether prothrombotic markers were associated with traditional risk factors, and clinical subgroups in a large population with stable CAD. The state of hypercoagulability was assessed by measures of sTF and TFPI, and F1+2, D-dimer and the CAT assay, the latter as marker of ex vivo thrombin generation.

We further wanted to explore the long-term effect of single clopidogrel as compared to single aspirin treatment on hypercoagulability in the same population, with the hypothesis that patients randomized to aspirin were less procoagulant.

## Methods

### Study subjects

A total of 1001 patients with angiographically verified stable CAD were enrolled in the ASpirin non-responsiveness and Clopidogrel clinical Endpoint Trial (ASCET) [11] at Oslo University Hospital, Ullevål, from 2004–2008. At inclusion all patients were on aspirin only and had been treated with aspirin only (160 mg/day) at least one week. Patients were thereafter randomized to either continous treatment with aspirin or to change to clopidogrel (75 mg/day) and the patients were further followed for two years. The present sub-study is based on data from baseline examinations in the total population and from the one-year follow up in a sub-set of 300 consecutively enrolled patients.

The study is in accordance with the principles of the declaration of Helsinki and is approved by the Regional Ethics Committee, and all patients have given written informed consent to participate. The study is registered at http://www.clinicaltrials.gov: NCT 00222261.

### Clinical subgroups

Within the population, previous myocardial infarction (MI) was recorded by patients’ medical files, and hypertension (HT) was defined as previously diagnosed and treated HT. Diabetes included individuals with treated type 2 diabetes (T2DM) and/or fasting glucose >7.0 mmol/L. The metabolic syndrome (MetS) was defined according to the American Heart Association/National Heart, Lung and Blood Institute [12]. Smoking habits were recorded as current smokers or not. Ex-smokers were included as non-smokers if they had quit smoking >3 months ago.

### Blood sampling

Venous blood samples were drawn in fasting condition between 08:00 and 10:30 am before intake of morning medication. Blood was collected in Vacutainer tubes containing 0.129 M trisodium citrate in dilution 1:10. The samples were stored on ice and centrifuged at 3000 x g for 20 min at 4°C within 1 hour. Plasma was kept frozen at −80°C until analyzed. Blood samples for routine analysis (glucose, HbA1C and lipids) were prepared and analyzed with conventional methods.

### Laboratory methods

Levels of sTF, TFPI, F1+2 and D-dimer were assessed by commercially available enzyme immunoassays; Imubind TF kit recognizing TF-apolipoprotein, sTF and TF-VII complexes (American Diagnostic Inc., Greenwich, Conn., USA), Asserachrom free and total TFPI antigen, recognizing full-length and full-length and truncated TFPI molecules associated to lipoproteins, respectively (Stago Diagnostica, Asniere, France), Enzygnost^®^ F1+2 (monoclonal) (Siemens, Marburg, Germany) and Asserachrom^®^ D-dimer (Stago Diagnostica). The coefficients of variation in our laboratory were 7.9, 5.6, 5.7 and 6.7%, respectively.

### The CAT assay

Ex vivo thrombin generation potential was investigated by the CAT assay, performed according to the manufacturer’s instruction (Thrombinoscope BV, Maastricht, The Netherlands). The method is described in detail elsewhere [13]. Briefly, 80μl thawed platelet poor plasma (PPP) was mixed with 20μl of a reagent containing recombinant relipidated TF and phospholipids, with the final concentrations of 5pM and 4μM, respectively. Reagents were provided from Thrombinoscope BV (Maastricht, The Netherlands). The reactions were performed in micro-titre wells (Immulon 2HB transparent U-bottom from Thermo Electron, Denmark) after automatic addition of a freshmade starting reagent containing CaCl_2_ (100 mM) and a thrombin specific fluorogenic substrate (Z-Gly-Gly-Arg-AMC) (2.5 mM) in Hepes buffer. The fluorescence intensity was recorded by the Fluoroskan Ascent^®^ microplate fluorometer (Thermo Fisher Scientific Oy, Vantaa, Finland). By simultaneous analysis of an inert thrombin calibrator with known thrombin activity, the software program (Thrombinoscope BV, version 3.0.0.29) is enabled to display the lag-time (LT, min), peak height (PeakH, nM), the endogenous thrombin potential (ETP, nM·min) and time to peak (ttPeak, min). The inter-assay coefficients of variation for the different parameters were 14.2%, 4.6%, 5.0% and 8.0%, respectively.

### Statistical methods

Continuous data on clinical characteristic of the population were mostly normally distributed, and data are presented as mean (SD) or proportions. As most of the measured variables were not normally distributed, data are presented as median values with 25, 75 percentiles. Univariate linear regression with log transformed values when appropriate, were used to analyse for associations between variables of thrombin generation and traditional risk factors. Linear regression models were also used to adjust for potential covariates in group comparisons.

Mann–Whitney U test or chi square test for categorical data were applied for group comparisons, and Wilcoxons test was used for within group changes. For difference in changes between the intervention groups, Mann–Whitney U test was used. P-values ≤0.05 were considered statistically significant. The statistical analyses were performed by SPSS Inc PASW statistics, version 18.

## Results

Clinical and laboratory characteristics of the total study population at baseline (n = 1001) are shown in Table 1. Mean age was 62 years and 22% of the patients were women. The patients were optimally treated, all receiving aspirin, 98% on statins, 76% on beta blockers and 50% on angiotensin converting enzyme inhibitors (ACEI) or angiotensin receptor blocker (ARB).

**Table 1:** Baseline characteristics of the total study population (n = 1001)

	
Age^1^ (years (range))	62 (36–81)
Male / Female (n (%))	783/218 (78/22)
Hypertension (n (%))	555 (55)
Metabolic syndrome (n (%))	244 (24)
Diabetes Mellitus (n (%))	200 (20)
Previous MI (n (%))	437 (44)
Current smokers (n (%))	204 (20)
BMI (kg/m^2^)	27.4 ± 3.7
Fasting glucose (mmol/L)	6.02 ± 1.82
HbA1C (%)	5.96 ±0.87
CRP (mg/L)	2.23 (1.03, 4.11)
Total cholesterol (mmol/L)	4.55 ±0.98
HDL cholesterol (mmol/L)	1.34 ±0.41
LDL cholesterol (mmol/L)	2.53 ± 0.83
Triglycerides (mmol/L) ^2^	1.29 (0.93, 1.82)
SBP (mmHg)	139 ±19
DBP (mmHg)	82 ±10
Medication (%)	
Aspirin	100
Statins	98
Beta-blockers	76
ACEI / ARB	50
Nitrates	22

### Associations

Associations between traditional risk factors and variables of thrombin generation are presented in Table 2. Age was significantly associated with F1+2 and D-dimer (p <0.001, both). Otherwise, only weak associations were found. For parameters in the CAT assay no significant associations were found (data not shown). Only results of ETP from the CAT assay are presented further.

**Table 2:** Associations between variables of thrombin generation and traditional risk factors in the total population

	**F1+2**		**D-dimer**		**ETP**	
	**beta**	**p-value**	**beta**	**p-value**	**beta**	**p-value**
Age	0.229	<0.001	0.417	<0.001	−0.026	0.424
Total cholesterol	0.102	0.001	0.051	0.110	0.060	0.062
HDL cholesterol	0.193	<0.001	0.131	<0.001	−0.133	<0.001
LDL cholesterol	0.050	0.120	0.048	0.134	0.089	0.006
Triglycerides	−0.058	0.066	−0.088	0.005	0.095	0.003
HbA1C	−0.063	0.047	0.044	0.164	0.077	0.016
Fasting glucose	−0.053	0.092	0.030	0.344	0.074	0.020
BMI	−0.125	<0.001	−0.043	0.177	0.072	0.024

Abbreviations: BMI: Body Mass Index.

### Thrombin generation in subgroups of CAD

The associations between different subgroups and sTF, free and total TFPI have previously been reported [14]. Markers of thrombin generation according to subgroups are presented in Table 3. Gender differences were observed for F1+2 and D-dimer, showing women to have higher levels, also after adjustment for covariates (age, BMI and HDL-cholesterol) (p <0.001 and p = 0.033, respectively). Smokers had significantly higher levels of ETP as compared to non-smokers (adjusted p = 0.014). In patients having MetS increased levels of ETP, but lower levels of F1+2 were observed. However, these differences were abolished after adjustments for covariates. No significant differences in any variables were observed with respect to having T2DM or not or whether they had experienced a MI or not.

**Table 3:** Variables of ex vivo and in vivo thrombin generation in subgroups of patients with stable CAD

	**ETP**	**F1+2**	**D-dimer**
	**(nM·min)**	**(pmol/L)**	**(ng/ml)**
Male (n = 771)	1439 (1240, 1599)	192 (146, 257)	381 (264, 595)
Female (n = 210)	1416 (1231, 1620)	251 (183, 356)	477 (338, 660)
p-value	0.745	<0.001 (<0.001^*^)	<0.001 (0.033^*^)
Smoking - (n = 779)	1423 (1225, 1590)	205 (152, 276)	394 (279, 613)
Smoking + (n = 202)	1466 (1291, 1633)	204 (153, 281)	419 (285, 609)
p-value	0.018 (0.014^*^)	0.789	0.605
MI - (n = 552)	1425 (1226, 1597)	202 (152, 277)	397 (273, 602)
MI + (n = 429)	1441 (1241, 1610)	206 (154, 277)	403 (285, 626)
p-value	0.325	0.567	0.428
T2DM - (n = 786)	1439 (1219, 1602)	207 (155, 278)	399 (278, 607)
T2DM + (n = 195)	1427 (1279, 1597)	192 (145, 263)	391 (283, 616)
p-value	0.671	0.079	0.667
MetS - (n = 739)	1420 (1208, 1581)	207 (157, 287)	399 (277, 614)
MetS + (n = 241)	1504 (1301, 1654)	187 (143, 251)	400 (282, 603)
p-value	<0.001 (0.283^*^)	0.002 (0.927^*^)	0.972
HT - (n = 436)	1430 (1224, 1603)	195 (148, 266)	377 (256, 583)
HT + (n = 545)	1440 (1246, 1601)	210 (156, 287)	419 (298, 625)
p-value	0.835	0.053 (0.532^*^)	0.01 (0.358^*^)
RAS inhib - (n = 491)	1440 (1210, 1619)	195 (145, 260)	383 (262, 561)
RAS inhib + (n = 483)	1436 (1245, 1590)	209 (159, 286)	419 (292, 658)
p-value	0.579	0.005 (0.013†)	0.001 (0.060†)
β-blockers - (n = 236)	1466 (1229, 1615)	200 (148, 258)	390 (267, 551)
β-blockers + (n = 737)	1433 (1239, 1600)	206 (153, 278)	401 (284, 626)
p-value	0.540	0.335	0.090

Medians (25, 75 percentiles) are given.

p-values refer to unadjusted differences between groups.

* Adjusted for age, BMI and HDL-cholesterol.

† Adjusted for age, BMI, HDL-cholesterol and creatinine.

Abbreviations: MI: myocardial infarction, T2DM: Type 2-diabetes mellitus, MetS: metabolic syndrome, HT: hypertension, RAS: renin angiotensin system

In univariate analyses significantly higher levels of F1+2 and D-dimer were observed in patients with HT, however, not statistically significant in multivariate analyses. A high number of patients were on ACEI and/or ARBS (n = 495) and in these patients compared to non-users, significantly higher levels of F1+2 and D-dimer were found. When adjusted for covariates, including creatinine, F1+2 levels were still significantly elevated (p = 0.013). No differences in any variables in patients using beta-blockers or not were observed.

### Influence of aspirin and clopidogrel on markers of coagulation

In the subset of patients (n = 300) of which 276 were available for re-investigation after 12 months, baseline characteristics did not differ between the randomized groups ( Additional file 1: Table S1). Of the measured variables significantly higher levels of sTF in the clopidogrel group were found at baseline (p <0.01) (Table 4). After 12 months intervention, significant reduction in F1+2 (p = 0.029) and ETP (p = 0.003) were observed within the aspirin group, whereas a slight, but statistically significant increase in sTF (p = 0.029) and free TFPI (p <0.001) were recorded. In the group randomized to clopidogrel, significantly reduced levels of ETP were found (p <0.001), while levels of free TFPI increased (p = 0.035). When comparing the changes between the groups, the reduction in F1+2 levels in the aspirin group was significantly different from the unchanged values in the clopidogrel group (p = 0.023). As for sTF the slight increase in the aspirin group was significantly different from the non-significant reduction in the clopidogrel group (p = 0.032). When taking the baseline levels into account by analyzing the difference in relative changes, the difference in change was still statistically significant (p = 0.042).

**Table 4:** Markers of coagulation at baseline (all patients on aspirin) and after 12 months continuing on aspirin or randomized to clopidogrel

	**ASPIRIN (n = 145)**			**CLOPIDOGREL (n = 131)**			
	**Baseline**	**12 months**	**p1**	**Baseline**	**12 months**	**p2**	**Δ p**
F1+2 (pmol/L)	216 (157, 282)	196 (142, 261)	0.029	201 (149, 279)	205 (158, 340)	0.298	0.023
D-dimer (ng/ml)	424 (271, 642)	434 (309, 661)	0.189	399 (284, 603)	409 (277, 583)	0.405	0.129
ETP (nM·min)	1502 (1308, 1626)	1397 (1148, 1571)	0.003	1492 (1281, 1676)	1366 (1165, 1584)	<0.001	0.687
sTF (pg/ml)	139 (91, 191)	143 (95, 206)	0.029	158^*^ (120, 218)	154 (118, 222)	0.384	0.042†
TFPI (free) (ng/ml)	15.3 (12.3, 17.9)	16.7 (13.0, 20.6)	<0.001	14.4 (11.4, 18.1)	15.3 (11.7, 18.6)	0.035	0.163
TFPI (total) (ng/ml)	68.4 (58.7, 76.6)	66.4 (58.6, 74.4)	0.190	66.1 (58.1, 76.6)	65.1 (57.9, 75.0)	0.130	0.864

Median values (25, 75 percentiles) are given.

^*^ refers to differences between groups at baseline (p <0.01).

p1 and p2 refer to differences from baseline to 12 months in the aspirin and clopidogrel groups, respectively. Δ p refer to difference in changes between groups from baseline to 12 months.

† refers to difference in relative changes from baseline to 12 months

## Discussion

In the present study on patients with stable CAD we could show that age was significantly associated to in vivo thrombin generation assessed by F1+2 and D-dimer. These markers were both higher in women than in men, and smokers had elevated levels of ex vivo thrombin generation measured by ETP, compared to non-smokers. Patients on ACEI and/or ARBS had significantly higher levels of F1+2 compared to non-users.

After one year treatment with either aspirin or clopidogrel, ETP levels were significantly reduced in both groups, and a significant reduction in F1+2 levels was observed on aspirin in contrast to on clopidogrel. The limited conformity between the investigated markers of in vivo and ex vivo thrombin generation, indicate that ongoing in vivo coagulation may not necessarily be compatible with a higher potential for ex vivo thrombin generation.

### Thrombin generation in subgroups of CAD

Among the traditional risk factors we found age to be strongest associated with the prothrombotic markers F1+2 and D-dimer. These results are in line with previous reports, demonstrated in a wide age span in healthy individuals [15]*.* Although these markers are known to be unspecific among the elderly, the risk of CVD in general is more pronounced with advancing age, partly due to an imbalance in the haemostatic system [16].

Regarding age and ex vivo thrombin generation (ETP) we could not find any association. This is in contrast to some previous reports, however, performed in quite different populations [17,18]. The discrepancy might be due to the rather large inter-individual variations reported for this assay, which may mask potential differences [7].

The women in our population were older compared to men, and they presented with higher levels of F1+2 and D-dimer, also after adjustment for age. ETP levels were, however not affected. This is in line with results from another similar group reporting elevated levels of prothrombotic markers in women compared to men [19]. Most of our women were postmenopausal, which also is associated with an adverse influence on traditional cardiovascular risk factors [20].

We demonstrated increased ex vivo thrombin generation assessed by ETP in smokers. To the best of our knowledge this has not been reported on before. In an Italian study it was shown a reduction in F1+2 and D-dimer levels after smoking cessation in individuals free from cardiovascular risk factors [21]. It is well-known that tobacco smoke increases platelet aggregability and it is discussed to have a direct toxic effect on the endothelium, including trigging haemostatic activation and thrombosis [22,23].

We could not demonstrate any increased activation of coagulation in patients with previous MI, T2DM or HT. According to several lines of evidence such patients are discussed to be more susceptible to atherothrombotic disease [24,25]. However, all our patients were very well medically treated, i.e. all patients were on aspirin and 98% on statins. Statins have been shown to reduce hypercoagulability, which may have masked any difference [26].

Somehow unexpected, patients on renin-angiotensin system (RAS) blockade, either with ACEI or ARB’s, had elevated levels of F1+2 compared to non-users, statistically significant also after correction for renal function. RAS blockade has been shown to be protective in different patient populations [27], possibly by improved haemodynamic stability, but also by prevention of angiotensin stimulated TF-expression and inhibition of fibrinolysis [28-30]. It has been shown significant correlations between components of the RAS, and levels of D-dimer, fibrinogen and plasminogen activator inhibitor type-1 in hypertensive patients, and it was suggested that plasma renin activity was the predominant component [31]. As renin is upstream of the levels of the blocade used, the increased levels of F1+2 might be discussed to be due to less consumption of renin*.*

### Effect of aspirin and clopidogrel on haemostatic markers

Data on anticoagulant effect of single antiplatelet treatment with either aspirin or clopidogrel, are sparse and conflicting. We demonstrated reduced thrombin generation, measured by ETP and F1+2, after long-term treatment with aspirin (160 mg/d), the latter being different from the changes on clopidogrel. This can be seen as contradictory to the CAPRIE trial in which clopidogrel was found to be slightly more effective compared to aspirin [32]. In this trial it should be noted that there were no difference in outcome between aspirin-or clopidogrel-treated patients entering the study with CAD, being present in all our patients. In addition to different mechanisms for inhibition, the patient population may thus also play a role for the effectiveness. In the present study, only effects on haemostatic markers were investigated. The effects on thrombin generation could potentially be ascribed to anti-inflammatory actions of aspirin. However, we have previously shown no difference in CRP-levels between the aspirin and clopidogrel groups in this cohort [9].

The decline in ETP could be explained by change in the assembly of pro- and anticoagulant factors after long-term treatment [13,33]. Activated platelets play a crucial role as a catalytic surface and a source for activated coagulation factor V, necessary for the large scale thrombin generation. During antiplatelet treatment this might decrease levels of F1+2, the most sensitive marker for in vivo thrombin generation [34]. Our results are in accordance with some previous reports, although, conflicting results exist [10,35].

Decreased thrombin generation by aspirin at the site of microvascular injury has furthermore been shown [36,37]. Several other mechanisms in the anticoagulant effect of aspirin have been discussed. The properties of aspirin to acetylate coagulation proteins and platelet receptors have been suggested to play a role [38]. It has moreover been shown decreased TF expression after treatment with aspirin in an experimental study related to reduced activity of the transcription factor NF-kappa B [39,40]. In our study, we observed a small but statistically significant increase in endogenous TF after 12 months on aspirin. This was not visualized in the CAT assay, which may be due to the relatively high dose of thrombin used in the assay.

In the group randomized to clopidogrel (75 mg/d) we observed a decline in thrombin generation after 12 months, however, only shown by ETP. Limited data on single long-term clopidogrel administration exist, but anti-thrombotic properties of clopidogrel has been shown at the site of microvascular injury in CAD patients [41]. In a rat modell a 23% decrease in ETP after clopidogrel ingestion, has also been shown [42]. On the contrary, several studies including dual anti-platelet treatment have not been able to demonstrate an additional beneficial effect of clopidogrel on top of aspirin on thrombin generation [43-45]. Blocking the ADP-pathway has nevertheless been shown to decrease collagen stimulated release of TF from platelet-leukocyte aggregates, and clopidogrel alone, as well as in combination with other antiplatelet agents, was reported to decrease circulating levels of TF [46,47]. This could not be confirmed in our study. This might be discussed along with the different TF methods used [47,48].

Free TFPI levels increased in both treatment groups. Whether this elevation is a compensatory mechanism to maintain the TF/TFPI balance is not known [49]. TFPI is a major determinant of ex vivo thrombin generation tests and might therefore explain the decrease in ETP levels, seen after one year in both groups [50].

## Conclusions

In summary, in the present population with stable CAD, we could demonstrate a more hypercoagulable profile among women, smokers and patients on RAS medication, assessed by the prothrombotic markers F1+2, D-dimer and ETP. Long-term antiplatelet treatment with aspirin alone seems to attenuate thrombin generation to a greater extent than with clopidogrel alone.

## Competing interests

The authors declare no competing interests.

## Authors’ contributions

VB conducted the study and was responsible for the blood sampling and the ETP, D-dimer and F1+2 analyses. VB also drafted and revised the manuscript. AAP contributed to the study protocol and acquired data. TBO performed the sTF and free- and total TFPI analyses in addition to interpretation of results. HA contributed to the study protocol and discussed the manuscript. IS conducted the study, contributed to the interpretation of results, drafted and revised the manuscript. All authors read and approved the final version of the manuscript.

## Authors information

VB: MSc

AAP: MD, PhD student

TBO: MSc, PhD student

HA: Professor, MD, PhD

IS: Professor, PhD

## Supplementary Material

Additional file 1 Table S1. Baseline characteristics according to the randomized groups. Values are mean (SD) and proportions if not otherwise stated. Click here for file

## References

[B1] World Health Organization Diabetes 2009 http://www.whoint/mediacentre/factsheets/fs312/en/

[B2] Libby P Ridker PM Maseri A Inflammation and atherosclerosis Circulation 2002 105 9 1135 1143 10.1161/hc0902.104353 11877368

[B3] Robbie L Libby P Inflammation and atherothrombosis Ann N Y Acad Sci 2001 947 167 179 11795264 10.1111/j.1749-6632.2001.tb03939.x

[B4] Broze GJ Jr Tissue factor pathway inhibitor and the current concept of blood coagulation Blood Coagul Fibrinolysis 1995 6 Suppl 1 S7 S13 7647225 10.1097/00001721-199506001-00002

[B5] Rapaport SI Rao LV Initiation and regulation of tissue factor-dependent blood coagulation Arterioscler Thromb 1992 12 10 1111 1121 10.1161/01.ATV.12.10.1111 1390583

[B6] Lwaleed BA Bass PS Tissue factor pathway inhibitor: structure, biology and involvement in disease J Pathol 2006 208 3 327 339 10.1002/path.1871 16261634

[B7] Hemker HC Al DR de Smedt E Beguin S Thrombin generation, a function test of the haemostatic-thrombotic system Thromb Haemost 2006 96 5 553 561 17080210

[B8] Ikonomidis I Andreotti F Economou E Stefanadis C Toutouzas P Nihoyannopoulos P Increased proinflammatory cytokines in patients with chronic stable angina and their reduction by aspirin Circulation 1999 100 8 793 798 10.1161/01.CIR.100.8.793 10458713

[B9] Solheim S Pettersen AA Arnesen H Seljeflot I No difference in the effects of clopidogrel and aspirin on inflammatory markers in patients with coronary heart disease Thromb Haemost 2006 96 5 660 664 17080224

[B10] Szczeklik A Krzanowski M Gora P Radwan J Antiplatelet drugs and generation of thrombin in clotting blood Blood 1992 80 8 2006 2011 1391958

[B11] Pettersen A-Å R SIAMAH Residual platelet reactivity on single aspirin treatment as related to clinical end points in patients with stable coronary artery disease. Results from the ASCET trial. 2012 Am Heart Assoc 2012 Ref Type: Generic10-1161/JAHA.112.000703 10.1161/JAHA.112.000703 PMC3487336 23130135

[B12] Grundy SM Cleeman JI Daniels SR Donato KA Eckel RH Franklin BA Diagnosis and management of the metabolic syndrome: an American Heart Association/National Heart, Lung, and Blood Institute scientific statement: Executive Summary Crit Pathw Cardiol 2005 4 4 198 203 10.1097/00132577-200512000-00018 18340209

[B13] Hemker HC Giesen P Al DR Regnault V De Smedt E Wagenvoord R Calibrated automated thrombin generation measurement in clotting plasma Pathophysiol Haemost Thromb 2003 33 1 4 15 10.1159/000071636 12853707

[B14] Opstad TB Pettersen AA Weiss T Arnesen H Seljeflot I Gender differences of polymorphisms in the TF and TFPI genes, as related to phenotypes in patients with coronary heart disease and type-2 diabetes Thromb J 2010 8 7 10.1186/1477-9560-8-7 20444258 PMC2882354

[B15] Cadroy Y Pierrejean D Fontan B Sie P Boneu B Influence of aging on the activity of the hemostatic system: prothrombin fragment 1 + 2, thrombin-antithrombin III complexes and D-dimers in 80 healthy subjects with age ranging from 20 to 94 years Nouv Rev Fr Hematol 1992 34 1 43 46 1523099

[B16] Lowe GD Rumley A Woodward M Morrison CE Philippou H Lane DA Epidemiology of coagulation factors, inhibitors and activation markers: the Third Glasgow MONICA Survey. I. Illustrative reference ranges by age, sex and hormone use Br J Haematol 1997 97 4 775 784 10.1046/j.1365-2141.1997.1222936.x 9217176

[B17] Haidl H Cimenti C Leschnik B Zach D Muntean W Age-dependency of thrombin generation measured by means of calibrated automated thrombography (CAT) Thromb Haemost 2006 95 5 772 775 16676066

[B18] van Hylckama Vlieg1 Christiansen SC Luddington R Cannegieter SC Rosendaal FR Baglin TP Elevated endogenous thrombin potential is associated with an increased risk of a first deep venous thrombosis but not with the risk of recurrence Br J Haematol 2007 138 6 769 774 10.1111/j.1365-2141.2007.06738.x 17760809

[B19] Ossei-Gerning N Wilson IJ Grant PJ Sex differences in coagulation and fibrinolysis in subjects with coronary artery disease Thromb Haemost 1998 79 4 736 740 9569183

[B20] Stevenson JC Crook D Godsland IF Influence of age and menopause on serum lipids and lipoproteins in healthy women Atherosclerosis 1993 98 1 83 90 10.1016/0021-9150(93)90225-J 8457253

[B21] Caponnetto P Russo C Di MA Morjaria JB Barton S Guarino F Circulating endothelial-coagulative activation markers after smoking cessation: a 12-month observational study Eur J Clin Invest 2011 41 6 616 626 10.1111/j.1365-2362.2010.02449.x 21198559

[B22] Cacciola RR Guarino F Polosa R Relevance of endothelial-haemostatic dysfunction in cigarette smoking Curr Med Chem 2007 14 17 1887 1892 10.2174/092986707781058832 17627524

[B23] Kimura S Nishinaga M Ozawa T Shimada K Thrombin generation as an acute effect of cigarette smoking Am Heart J 1994 128 1 7 11 10.1016/0002-8703(94)90003-5 8017287

[B24] Celi A Cianchetti S Dell’Omo G Pedrinelli R Angiotensin II, tissue factor and the thrombotic paradox of hypertension Expert Rev Cardiovasc Ther 2010 8 12 1723 1729 10.1586/erc.10.161 21108554

[B25] Grant PJ Diabetes mellitus as a prothrombotic condition J Intern Med 2007 262 2 157 172 10.1111/j.1365-2796.2007.01824.x 17645584

[B26] Koh KK Quon MJ Waclawiw MA Are statins effective for simultaneously treating dyslipidemias and hypertension? Atherosclerosis 2008 196 1 1 8 10.1016/j.atherosclerosis.2007.06.006 17662294 PMC2742669

[B27] Vijayaraghavan K Deedwania P Renin-angiotensin-aldosterone blockade for cardiovascular disease prevention Cardiol Clin 2011 29 1 137 156 10.1016/j.ccl.2010.11.003 21257105

[B28] Brown NJ Vaughan DE Prothrombotic effects of angiotensin Adv Intern Med 2000 45 419 429 10635057

[B29] Celi A Del FA Cianchetti S Pedrinelli R Tissue factor modulation by Angiotensin II: a clue to a better understanding of the cardiovascular effects of renin-angiotensin system blockade? Endocr Metab Immune Disord Drug Targets 2008 8 4 308 313 10.2174/187153008786848259 19075785

[B30] Vaughan DE The renin-angiotensin system and fibrinolysis Am J Cardiol 1997 79 5A 12 16 9127616 10.1016/s0002-9149(97)00124-0

[B31] Sechi LA Novello M Colussi G Di FA Chiuch A Nadalini E Relationship of plasma renin with a prothrombotic state in hypertension: relevance for organ damage Am J Hypertens 2008 21 12 1347 1353 10.1038/ajh.2008.293 18948960

[B32] CAPRIE Steering Committee A randomised, blinded, trial of clopidogrel versus aspirin in patients at risk of ischaemic events (CAPRIE) Lancet 1996 348 9038 1329 1339 8918275 10.1016/s0140-6736(96)09457-3

[B33] Beguin S Keularts I On the coagulation of platelet-rich plasma Physiological mechanism and pharmacological consequences. Haemostasis 1999 29 1 50 57 10.1159/000022460 10494034

[B34] Bauer KA Laboratory markers of coagulation activation Arch Pathol Lab Med 1993 117 1 71 77 8418767

[B35] Brodin E Seljeflot I Arnesen H Hurlen M Appelbom H Hansen JB Endogenous thrombin potential (ETP) in plasma from patients with AMI during antithrombotic treatment Thromb Res 2009 123 4 573 579 10.1016/j.thromres.2008.03.018 18474393

[B36] Kyrle PA Westwick J Scully MF Kakkar VV Lewis GP Investigation of the interaction of blood platelets with the coagulation system at the site of plug formation in vivo in man–effect of low-dose aspirin Thromb Haemost 1987 57 1 62 66 2954260

[B37] Undas A Undas R Musial J Szczeklik A A low dose of aspirin (75 mg/day) lowers thrombin generation to a similar extent as a high dose of aspirin (300 mg/day) Blood Coagul Fibrinolysis 2000 11 3 231 234 10870801

[B38] Undas A Brummel-Ziedins KE Mann KG Antithrombotic properties of aspirin and resistance to aspirin: beyond strictly antiplatelet actions Blood 2007 109 6 2285 2292 10.1182/blood-2006-01-010645 17148593 PMC1852201

[B39] Matetzky S Tani S Kangavari S Dimayuga P Yano J Xu H Smoking increases tissue factor expression in atherosclerotic plaques: implications for plaque thrombogenicity Circulation 2000 102 6 602 604 10.1161/01.CIR.102.6.602 10931797

[B40] Osnes LT Foss KB Joo GB Okkenhaug C Westvik AB Ovstebo R Acetylsalicylic acid and sodium salicylate inhibit LPS-induced NF-kappa B/c-Rel nuclear translocation, and synthesis of tissue factor (TF) and tumor necrosis factor alfa (TNF-alpha) in human monocytes Thromb Haemost 1996 76 6 970 976 8972019

[B41] Dropinski J Musial J Jakiela B Wegrzyn W Sanak M Szczeklik A Anti-thrombotic action of clopidogrel and P1(A1/A2) polymorphism of beta3 integrin in patients with coronary artery disease not being treated with aspirin Thromb Haemost 2005 94 6 1300 1305 16411409

[B42] Herault JP Dol F Gaich C Bernat A Herbert JM Effect of clopidogrel on thrombin generation in platelet-rich plasma in the rat Thromb Haemost 1999 81 6 957 960 10404775

[B43] Eikelboom JW Weitz JI Budaj A Zhao F Copland I Maciejewski P Clopidogrel does not suppress blood markers of coagulation activation in aspirin-treated patients with non-ST-elevation acute coronary syndromes Eur Heart J 2002 23 22 1771 1779 10.1053/euhj.2000.3234 12419297

[B44] Kringen MK Narum S Lygren I Seljeflot I Sandset PM Troseid AM Reduced platelet function and role of drugs in acute gastrointestinal bleeding Basic Clin Pharmacol Toxicol 2011 108 3 194 201 10.1111/j.1742-7843.2010.00643.x 21118353

[B45] Undas A Stepien E Branicka A Wolkow P Zmudka K Tracz W Thrombin formation and platelet activation at the site of vascular injury in patients with coronary artery disease treated with clopidogrel combined with aspirin Kardiol Pol 2009 67 6 591 598 19618315

[B46] Leon C Alex M Klocke A Morgenstern E Moosbauer C Eckly A Platelet ADP receptors contribute to the initiation of intravascular coagulation Blood 2004 103 2 594 600 10.1182/blood-2003-05-1385 12969982

[B47] Rao AK Vaidyula VR Bagga S Jalagadugula G Gaughan J Wilhite DB Effect of antiplatelet agents clopidogrel, aspirin, and cilostazol on circulating tissue factor procoagulant activity in patients with peripheral arterial disease Thromb Haemost 2006 96 6 738 743 17139367

[B48] Parhami-Seren B Butenas S Krudysz-Amblo J Mann KG Immunologic quantitation of tissue factors J Thromb Haemost 2006 4 8 1747 1755 10.1111/j.1538-7836.2006.02000.x 16879217

[B49] Morange PE Renucci JF Charles MA Aillaud MF Giraud F Grimaux M Plasma levels of free and total TFPI, relationship with cardiovascular risk factors and endothelial cell markers Thromb Haemost 2001 85 6 999 1003 11434709

[B50] Winckers K Siegerink B Duckers C Maurissen LF Tans G Castoldi E Increased tissue factor pathway inhibitor activity is associated with myocardial infarction in young women: results from the RATIO study J Thromb Haemost 2011 9 11 2243 2250 10.1111/j.1538-7836.2011.04497.x 21895962

