# Supplementary material for: Markers of hypercoagulability in CAD patients. Effects of single aspirin and clopidogrel treatment
Source: Thromb J. 2012 Aug 10;10:12. doi: 10.1186/1477-9560-10-12 (PMC3552672; doi:10.1186/1477-9560-10-12)
Supplement: Additional file 1 — Table S1. Baseline characteristics according to the randomized groups. Values are mean (SD) and proportions if not otherwise stated. [file 1477-9560-10-12-S1.doc]

Supplementary Table Baseline characteristics according to the randomized groups. Values are mean (SD) and proportions if not otherwise stated.

|  | Aspirin | Clopidogrel | p-value |
| --- | --- | --- | --- |
| Age^1^ (years (range)) | 62 (39, 81) | 61 (42, 80) | 0.750 |
| Male / Female (%) | 80/20 | 80/20 | 0.908 |
| Hypertension (%) | 47 | 57 | 0.063 |
| Previous MI (%) | 53 | 53 | 0.897 |
| Diabetes Mellitus (%) | 24 | 26 | 0.689 |
| MetS (%) | 31 | 26 | 0.382 |
| Smoke (%) | 23 | 21 | 0.770 |
| BMI (kg/m^2^) | 28.2 ± 4.2 | 27.3 ± 3.5 | 0.084 |
| Fasting glucose (mmol/L) | 6.25 ± 2.25 | 6.41 ± 2.22 | 0.617 |
| HbA1C (%) | 6.26 ± 1.08 | 6.31 ± 0.96 | 0.287 |
| Total cholesterol (mmol/L) | 4.63 ± 0.85 | 4.68 ± 1.04 | 0.801 |
| HDL cholesterol (mmol/L) | 1.26 ± 0.37 | 1.32 ± 0.39 | 0.136 |
| LDL cholesterol (mmol/L) | 2.66 ± 0.75 | 2.62 ± 0.74 | 0.678 |
| Triglycerides (mmol/L) ^2^ | 1.44 (0.94, 1.96) | 1.40 (0.97, 1.85) | 0.508 |
| SBP (mmHg) | 142 ± 20 | 144 ± 23 | 0.856 |
| DBP (mmHg) | 82 ± 10 | 84 ± 10 | 0.188 |
| Medication (%) |  |  |  |
| Statins | 97 | 97 | 0.705 |
| Beta-blockers | 80 | 78 | 0.688 |
| ACEI | 30 | 36 | 0.252 |
| ARBs | 22 | 20 | 0.651 |
| Nitrates | 30 | 26 | 0.452 |

^1^ mean (range)

^2^ median (25, 75 percentiles)

p-values refer to differences between randomized groups

Abbreviations: MI: myocardial infarction, MetS: metabolic syndrome, BMI: Body Mass Index, SBP: systolic blood pressure, DBP: diastolic blood pressure, ACEI: angiotensin converting enzyme inhibitor, ARB: angiotensin receptor blocker.
